# FoldX as Protein Engineering Tool: Better Than Random Based Approaches?

**DOI:** 10.1016/j.csbj.2018.01.002

**Published:** 2018-02-03

**Authors:** Oliver Buß, Jens Rudat, Katrin Ochsenreither

**Affiliations:** Institute of Process Engineering in Life Sciences, Section II: Technical Biology, Karlsruhe Institute of Technology, Karlsruhe, Germany

**Keywords:** FoldX, Fold-X, Thermostability, Protein stabilization, Protein engineering, Enzyme engineering

## Abstract

Improving protein stability is an important goal for basic research as well as for clinical and industrial applications but no commonly accepted and widely used strategy for efficient engineering is known. Beside random approaches like error prone PCR or physical techniques to stabilize proteins, e.g. by immobilization, in silico approaches are gaining more attention to apply target-oriented mutagenesis. In this review different algorithms for the prediction of beneficial mutation sites to enhance protein stability are summarized and the advantages and disadvantages of FoldX are highlighted. The question whether the prediction of mutation sites by the algorithm FoldX is more accurate than random based approaches is addressed.

## Graphical Abstract

**Figure:**
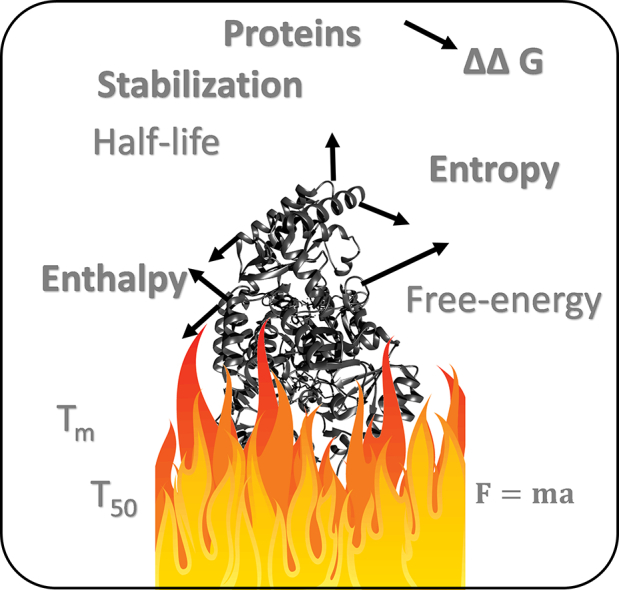

## 1 Introduction

Increasing protein stability is a desirable goal for different life science purposes, this includes design of therapeutic proteins like antibodies, human cell biology and biotechnology. It is expected that such improvements result in lower process costs and in enhanced long-term stability of the applied proteins. Enhanced protein stability in general can be achieved due to various factors, e.g. by increasing thermostability, salt tolerance or tolerance towards organic solvents, and consequently, involves different bioinformatics approaches. The emphasis for application of proteins for medical and chemical purposes is focused on the fields of biosensors (e.g. blood sugar test strips [1]), biomedical drugs (e.g. antibodies against cancer cells [2]) or on the synthesis of complex as well as chiral substances for food (e.g. high fructose corn syrup [3]) and pharmaceutical industry (e.g. sitagliptin [4]) [5]. Obviously biosensors for medical use assisting to diagnose several diseases like breast cancer [6], diabetes [7] or infectious diseases [8] have to be functional and reliable for a defined period of time. It seems, for example, to be beneficial to gain more thermostable antibodies for treatment of cancer diseases [9]. Furthermore, for the synthesis processes of drugs and pharmaceutically relevant intermediates, applied enzymes have to be active and functional for long batch times to prevent drastic increases in costs per unit of product [[10], [11], [12]] [13]. For industrial enzymes improved stability against heat, solvents and other relevant process parameters, e.g. acidic or basic pH, often becomes crucial [14]. In addition, improved thermostability of enzymes might prevent thermal inactivation and conformational changes at higher reaction temperature, which could in turn be beneficial to raise turnover rates and substrate concentrations [[15], [16], [17], [18], [19]]. According to the Q_10_-rule of thumb, biological systems and enzymes tend to have a Q-factor of 2, i.e. a temperature increase of about 10 K results in doubling the reaction rate [20,21]. Contrary, stabilization also can lead to more rigid enzymes, which are less active at the same temperature, but show the same activity at elevated temperatures. This can be observed when enzymes from hyperthermophilic and mesophilic sources are compared with respect to their reaction rates [22]. A thermostabilized enzyme might be less active at a certain temperature, but longer active at higher temperatures, which allows applying the Q_10_-rule on the condition that the activity can be maintained for longer time periods at elevated temperatures [23,24]. However, it is also possible that a thermostabilized enzyme is not impaired in activity at moderate temperatures and is even more active at higher temperatures [[25], [26], [27], [28], [29]]. Arnold et al. demonstrated that an enzyme can be simultaneously developed towards higher stability and activity [24,30,31,45].

Protein denaturation and degradation due to both heat and solvents are based on the same protein unfolding processes. The most important forces for protein stability, which are relevant targets for the improvement of protein stability, are intramolecular interactions, i.e. disulfide bridges, ion interactions, hydrogen bonds, hydrophobic interactions and core packings [22]. The rigidity and flexibility of proteins seem to be the key parameters [33] and both can be influenced by using immobilization techniques or enzymatic engineering in order to expand the durability of protein applications.

The well-known technique to immobilize proteins to gain stabilized proteins is applied for antibody to increase the thermostability [34,35]. Beside the improvement of thermostability using immobilization techniques, directed evolution is an alternative approach, but the existence of a robust high-throughput screening assay for the selected protein is an important prerequisite [11,[36], [37], [38], [39], [40], [41]]. For enzymes, activity can be used as parameter for functionality at elevated temperatures, but for non-catalyzing proteins a more sophisticated assay or even protein purification is necessary. Furthermore, the number of necessary protein variants, created by using e.g. error prone PCR or other techniques, is mostly about 10^3^ to 10^5^ and even higher. However, in case of enzymes, selection might easily be performed by heating up unpurified crude extracts from cells [42]. Using this technique, protein melting temperature T_m_ can be improved in the best screenings by far >10 °C [[42], [43], [44], [45], [46], [47]]. The artificial evolution approach can result in a 140-fold increase in long-term enzymatic activity like demonstrated for the alkaline pectate lyase [48]. Also for antibodies or antibody fragments evolutionary approaches can be used [49]. For example, the protein melting temperature of a human antibody domain was improved by >10 °C [50].

Directed evolution can be a successful strategy but might not be applicable at any time, especially when missing a high-throughput screening or protein purification for stability measurements is necessary. Therefore, this mini-review focusses on protein/enzyme engineering for thermostabilization using structure guided site-directed mutagenesis. This strategy helps to reduce screening effort and also costs, which is an issue in large screenings. Furthermore, we selected the popular FoldX algorithm and would like to answer the question: how powerful is FoldX for common protein stability improvements? FoldX is a frequently used algorithm and many studies about protein stabilization experiments are described in literature. A second reason is the user-friendliness of FoldX, because it can easily be used as plugin in the protein structure visualizer YASARA [51]. In contrast to other, command-line based in silico approaches, which are without graphical interface, scientists not familiar with programming languages like python, Java, R-script and so on and hide a larger workload for these kind of approaches.

### 1.1 Computational Approaches for Stability Engineering

Besides FoldX, several other algorithms used for site-directed mutagenesis are also known aiming at different inter- and intra-protein interactions. One target is the introduction of artificial disulfide bridges into proteins. As a covalent bond, a disulfide bridge is a strong physical force which helps to stabilize the 3D-structure within a protein chain or between monomers raising the protein melting temperature (T_m_) up to 30 °C, and can achieve an increase of thermal stability by >40% at distinct temperature levels [[52], [53], [54]]. However, introduction of disulfide bridges can also lower the T_m_ up to −2.4 °C [55]. Starting with the protein structure as basis for molecular dynamic simulations and energy calculations, amino acid positions can be selected which are potentially suitable for engineering of disulfide bridges. However, these approaches need profound understanding of different prediction and calculation software, often without graphical interfaces [56]. Two examples are the algorithms for fast recognition of disulfide mutation sites FRESCO and the open access webtool “Disulfide by Design 2” (DD2), but only DD2 can be easily used with graphical interface [[57], [58], [59]]. Using FRESCO, a temperature improvement of 35 °C was achieved due to the combination of single disulfide bridges. Jo et al. increased the T_m_ of the α-type carbonic anhydrase by 7.8 °C due to an introduction of a disulfide bond efficiently predicted by DD2 [60]. Albeit the promising examples, it has to be mentioned, that the extensive FRESCO strategy cannot be understood as an end-user script, but more or less as a blue script for improving thermostability. Wijma et al. further improved FRESCO by integrating FoldX and Rosetta as additional energy improvement tools and combined these results with Dynamic Disulfide Discovery algorithm based on molecular dynamic simulations [57,145]. After in silico elimination of less stable variants, they expressed, tested and combined beneficial point mutation sites and disulfide bonds to gain two variants with drastically increased T_m_ of 34.6 and 35.5 °C, respectively. However, this strategy is very extensive and many point mutations have to be tested and combined.

Beside the possible de novo design of disulfide bridges, further computational methods like helix dipole stabilization or core repacking exist. Core repacking aims only at the core region of proteins to increase hydrophobic interactions. Vlassi et al. showed that a reduction of hydrophobic interaction decreases the protein stability [61] and computational tools like RosettaDesign and Monte Carlo simulations are used for the optimization process [[62], [63], [64]]. Adapted and automated RosettaDesign framework for repacking are available, but profound programming capabilities are needed for applying [64]. In contrast, helix dipole stabilization methods lead to improvements of molecular interactions at the end of helices, which can also result in drastically increased T_m_ by >30 °C [65,66]. However, for this strategy elaborate electrostatic calculations and molecular simulations are needed to select mutation sites. Beside these strategies, consensus sequences can also help to improve protein stability using multiple sequence alignments. In so-called consensus guided mutagenesis, sequences are compared according to their amino acid frequencies to elucidate consensus sequences. Replacing amino acid residues at certain positions with the most prevalent ones often result in highly beneficial energy improvements stabilizing proteins [[67], [68], [69], [70]]. Huang et al. demonstrated that by using consensus approach it was possible to improve the stability of the reductase CgKR1 T_50_^15^ (temperature at which the enzyme activity is halved within 15 min) by >10 °C [71].

## 2 Un/folding Energy Algorithms

At least 22 standalone calculation tools are described for the prediction of beneficial single and multiple point mutation sites to reduce the Gibbs free energy of proteins. The broad diversity of these standalone software was reviewed by Modarres et al. and beside the mentioned FoldX algorithm, other tools like PoPMuSiC, CUPSAT, ZEMu, iRDP web server or SDM were mentioned [[72], [73], [74], [75]]. These calculation tools are structure or sequence dependent and use energy calculation functions or machine learning algorithms. Also databases collecting changes in protein stability (e.g. for Gibbs free energy changes and melting temperatures) are available like ProTherm (others are e.g. MODEL, DSBASE), but it should be mentioned that 70% of the logged mutations are destabilizing which leads to unintended biases [73,76]. Beside the more popular algorithms others are published like mCSM, BeAtMuSiC and ENCoM using different calculation approaches [[77], [78], [79]]. Moreover, it is also possible to use crystallographic data gained by X-ray analysis of protein structure. The B-factor is an indicator for the flexibility of positions within the protein. Reetz et al. used this factor for increasing protein stability [80].

### 2.1 FoldX

Considering the diversity of available algorithms, it seems to be very difficult to choose an efficient tool for protein stabilization. In this review we concentrated on the force field algorithm FoldX, which we have used by ourselves to create a more stable ω-transaminase [81]. The force field algorithm, which was originally created by Guerois et al. became popular as webtool in 2005 by Schymkowitz et al. and was refined to the currently last version FoldX 4.0 [[82], [83], [84]].

The software package FoldX includes different subroutines e.g. RepairPDB, BuildModel, PrintNetworks, AnalyseComplex, stability and so on. For example the repair function of FoldX reduces the energy content of a protein-structure model to a minimum by rearranging side chains and the function BuildModel introduces mutations and optimizes the structure of the new protein variant. The energy function of FoldX is only able to calculate the energy difference in accurate manner between the wildtype and a variant of the protein [83].

$$\mathrm{\Delta \Delta G}={\mathrm{\Delta G}}_{\text{wildtype}}-{\mathrm{\Delta G}}_{\text{variant}}\;\left[\text{kcal}\;{\mathrm{mol}}^{-1}\right]$$

FoldX is also able to calculate total energies of objects, but this function is only valid to predict, whether a problem with the structure is given or not. The total energy results are not able to predict experimental results [51,83]. The core function of FoldX, the empirical force field algorithm, is based on free energy (ΔG) terms aiming to calculate the change of ΔG in kcal mol^−1^ (Eq. (1)). This equation includes terms for polar and hydrophobic desolvation or hydrogen bond energy ΔG_wb_ of a protein interacting with solvent and within the protein chain. Increased protein rigidity works against entropy and consequently, results in entropy costs.

$$\begin{array}{l} \mathrm{\Delta G}=a\;{\mathrm{\Delta G}}_{\mathrm{vdw}}+b\;{\mathrm{\Delta G}}_{\text{solvH}}+c\;{\mathrm{\Delta G}}_{\text{solvP}}+d\;{\mathrm{\Delta G}}_{\mathrm{wb}}+e\;{\mathrm{\Delta G}}_{\text{hbond}}\\ +f\;{\mathrm{\Delta G}}_{\mathrm{el}}+g\;{\mathrm{\Delta G}}_{\mathrm{kon}}+h\;{\mathrm{T\Delta S}}_{\mathrm{mc}}+k\;{\mathrm{T\Delta S}}_{\mathrm{mc}}+l\;{\mathrm{\Delta G}}_{\text{clash}} \end{array}$$

Furthermore the energy algorithm also addresses the free energy change at protein interfaces of oligomeric proteins. This term is mainly ΔG_kon_ which calculates the electrostatic contribution of interactions at interfaces [83]. The parameters which are important for the energy calculation were determined in laboratory experiments, e.g. for amino acid residues and explored on protein chains. Beside this distinct parameters the letters of the total energy equation, a to l, represent the weights of separate terms [83]. The algorithm works with optimal accuracy when the hypothetical unfolding energy difference of the hypothetical energy from a wild-type variant is determined in comparison to a mutated protein. For this purpose, FoldX uses the 3D structure to calculate the hypothetical unfolding energy. The algorithm was first implemented as free available web server tool and is now a commercially available software, which can be used free of charge for academic purposes. As a prerequisite, a highly resolved crystal structure is necessary to calculate the energy changes for site-directed mutagenesis experiments. Users can also automate the calculations e.g. by using the programming code Python to calculate whole protein amino acid exchanges at every distinct position [85,86]. Furthermore, FoldX shows very good performance with respect to calculation time even on single core computers. Compared to e.g. ZEMu, FoldX needs only half the time for calculating single site mutations (calculated on one single processor) and is faster than RosettaDDG [75,87]. As mentioned earlier, it can be used with a graphical user interface as plugin tool in YASARA, which opens FoldX towards a broad community of researchers.

### 2.2 FoldX-applications

FoldX was applied for different stability tests, especially when protein design was performed to predict whether distinct mutations are destabilizing. Therefore FoldX shows to be beneficial for different approaches and is not strictly limited to a distinct function. Moreover the peptides, individual domains and multi-domain proteins can be addressed for experiments [88,89]. The algorithm has been used to explain and predict stability improvements when designing solvent stable enzymes. The group of U. Schwaneberg designed a laccase with improved resistance in ionic liquids for using hardly soluble lignin lysates and increased tolerance towards high molarity of salts [90]. Beside its suitability for protein energy calculations, it is also possible to calculate the energy changes of DNA-protein interactions [91]. Furthermore, FoldX is implemented in a lot of approaches like Fireprot, FRESCO, TANGO or in combination with Voronoia 1.0. Voronoia helps to engineer protein core packing and is based on energy calculations using FoldX as force field algorithm [92,93]. The program FRESCO (Framework for Rapid Enzyme Stabilization by Computational libraries) joins Rosetta with FoldX energy calculations and combines single point mutations with disulfide predictions for drastic energy improvements of enzymes [57]. The direct alternative to FoldX is the Rosetta energy algorithm. It was shown, that Rosetta predicts other possible mutation sites than FoldX for energy improvements, but only 25% of all mutations were predicted by both algorithms for the same protein [57]. Additionally, the authors of this work excluded 52% of the selected mutations manually, e.g. excluding hydrophobic mutations on surface exposed sites and mutations to a proline residue or a proline residue to a non-proline residue. At the end around 65% of the predicted mutation sites were calculated by FoldX and thereby 35% of all predicted sites were discarded. Voronoia in combination with FoldX helps to predict and to explain why hydrophobic interactions in the core region can have a huge impact on protein stability, as it was demonstrated for the thermophilic lipase T1 [93]. Another approach is TANGO, which helps to predict the aggregation of proteins and, in combination with FoldX, is a powerful tool for the investigation of predicted mutations regarding solubility, e.g. protective site-directed mutations for the Alzheimer's αβ peptide [83,94,95]. Furthermore, FoldX can also support protein design. For engineering the zinc-finger nuclease, FoldX was used as prediction algorithm to detect if the binding energy of a distinct DNA-sequence was increased or decreased [96]. Also, FoldX can help to estimate protein-protein binding energy and resulting stabilities of protein complexes. Szczepek et al. redesigned the interface between dimeric zinc finger nucleases using FoldX as prediction tool [97]. After deeper in silico calculations, only 9.3% of predicted variants were expressed and proved to be beneficial for stability [97]. Considering these and other experiments the performance for FoldX should be critically evaluated.

Therefore, we gathered FoldX experiments and analyzed available publications if FoldX was helpful for increasing protein stability (Table 1). In general, the amount of standalone FoldX calculations for protein stability improvement in literature is relatively low compared to approaches, which are using FoldX as an additional tool for stability calculation. Furthermore, FoldX is often only used as algorithm for explanations of the impact of mutagenesis in proteins with respect to stability or towards predictions of protein-protein or protein-DNA binding. Therefore, in Table 1 only mutations with effects based on FoldX predictions are pointed out, even when authors used additional calculation tools. When no pre-selection of distinct protein sites are indicated, a complete calculation of every position in the protein was performed. In this case, every amino acid was exchanged with the 19 standard amino acid residues. This calculation setup results very fast in high numbers of predicted variants. One criterion for excluding many variants is to set an energy barrier for ΔΔG between −0.75 and −5 kcal mol^−1^ for stabilizing mutations and for destabilizing mutations of >+1 kcal mol^−1^ in accordance to the Gaussian distribution of FoldX predictions (SD for FoldX 1.78 kcal mol^−1^ [95]) [98]. After this pre-selection a large number of variants can be excluded. Furthermore, mutations nearby active sites, proline residue mutations or variants which seem to be critical for protein structure can be also excluded. In addition to manual exclusion of variants, also MD-simulations can be performed to exclude more variants. Aiming to indicate the grade of improvement, protein melting temperature T_m_ or half-life activity is frequently used. The largest positive changes in stability were reached for the T1 lipase, phosphotriesterase, Flavin-mononucleotide-based-fluorescent-protein and for the haloalcohol dehalogenase ranging from 8 up to 13 °C using single site mutations [99]. However, FoldX also allows prediction of destabilizing mutations, which were performed very accurately for the thermoalkalophilic lipase with a negative ΔT_m_ of 10 °C. Noticeably, stabilizing predictions are useful for biotechnology and are therefore mentioned in studies with biotechnological background, whereas destabilizing predictions seem to be more applicable for human disease studies [95]. Beside mere stability studies, also protein design was performed towards specific enzyme-DNA binding or antibody-antigen binding, which can reduce the size of antibody libraries for distinct antigen targets. Moreover, FoldX can also be used to adapt or to select mutations to increase stability in ionic liquids. For this, FoldX calculations were performed with increasing salt concentration during the simulations, but currently no further experiments towards ionic liquid improvement can be found. In literature also numerous examples can be found dealing with diseases caused by protein mutations. These investigations aim to prove whether human proteins are less stable with a mutation compared to the wild-type or might interact differently with other proteins [[100], [101], [102]].

**Table 1:** Summary of different FoldX applications for single point mutations regarding stability and ligand binding. The changes achieved i.e. T_m_ is listed for changes in protein melting temperature. ΔΔG displays the change in free energy by mutation/design of proteins. “Criteria” describes the settings for experiments. “Cut-off” means, that the authors excluded those indicated FoldX predictions (with a higher or lower ΔΔG) from further experiments. ΔΔG is defined as: ΔΔG = ΔG^fold^(mutation) − ΔG^fold^(wild type).

Aim of the study	Protein/Source	Criteria	Number of tested predictions	Number of correct predictions	Greatest impact	Resolution crystal structure	Ref.
Enzyme stabilization	Endoglucanase (*Hypocrea jecorina*)	Cut off value < ΔΔG −1.75 kcal mol^−1^	43	6	Stabilization (ΔT_m_ = 3.2 °C)	1.62 Å	[65]
	Phosphotriesterase (*Pseudomonas oleovorans*)	Cut off value < ΔΔG −0.72 kcal mol^−1^	52	32	Stabilization (ΔT_m_ = 8.6 °C)	2.25 Å	[103]
	T1 Lipase (*Geobacillus zalihae*)	One mutation site was selected and exchanged against Val, Ile, Met, Phe, Trp compared to wild type	7	1	Stabilization (+ΔT_opt-_ = 10 °C)	1.5 Å	[93]
	Thermoalkalophilic lipase (*Bacillus thermocatenulatus*)	3 sites preselected and amino acids were exchanged against Phe, Try and Trp.	9	2	Destabilizing variants (ΔT_m_ = −10 °C)	2.0 Å	[104]
	Haloalkane dehalogenase (WT and one mutant) *Sphingomonas paucimobilis*	Cut off value < ΔΔG −0.84 kcal mol^−1^ + visual inspection and MD-simulation	<150	5	Stabilization (ΔT_m_ = 3 °C)	0.95 Å	[105]
	Limonene-1,2-epoxide hydrolase (*Rhodococcus erythropolis*)	Cut off (ΔΔG < −1.2 kcal mol^−1^) performed additionally further pre-selection	21	6	Stabilization ΔT_m_ = 6 °C	1.2 Å	[57]
	Cellobiohydrolase (*Hypocrea jecorina*)	43 mutations selected (ΔΔG < −0.75 kcal mol^−1^)	43	10	Stabilization ΔT_m_ = 0.7 °C	2.35 Å	[70]
	*ω*-Transaminase (*Variovorax paradoxus*)	Cut off value ΔΔG < −6.5 kcal mol^−1^	11	3	ΔT_m_ = 4 °C	2.28 Å	[81]
	Amine transaminase (*Aspergillus terreus*)	B-factor was used as pre-filter for FoldX predictions towards stabilization	19	6	Stabilization ΔT_1/2_^10min^ = 3.5^°^C	1.63 Å	[106]
	Laccase (*Trametes versicolor* and fungus PM1)	Molecular dynamic averaged structures were used for FoldX calculation	11	Standard deviation max. ΔΔG < 1 kcal mol^−1^	ΔT_m_ = 3–5 °C	2.4 Å	[107]
	Haloalcohol dehalogenase (*Agrobacterium tumefaciens*)	Cut off (ΔΔG < −1.2 kcal mol^−1^) 775 mutants were predicted by FoldX and reduced using Rosetta-dgg and MD-simulation	55	29	Stabilizing ΔT_m_ = 13 °C	1.9 Å	[99]
	Chalcone synthase (*Physcomitrella patens*)	Calculation of total energy ΔG = −63 up to 67 kcal mol^−1^ Single site variant	19	2	1 variant showed high thermal stability	Homology modeling	[108]
	Carbonyl reductase (*Streptomyces coelicolor*)	Variants with ΔΔG < −4 kJ mol^−1^ were selected	3	1	Stabilization of ΔT_50_^15^ = 1.3^°^C	1.6 Å	[109]
	Peptide Amidase (*Stenotrophomonas maltophilia*)	Cut off value ΔΔG < −5 kJ mol^−1^	44	6	Stabilizing ΔT_m_ = 6 °C	1.8 Å	[110]
Enzyme stabilization and comparison to other tools	Penicillin G acylase (*Escherichia coli*)	Not reported	21	8	–	1.9 Å	[111]
Enzyme destabilization	Triosephosphate isomerase (*Saccharomyces cerevisiae*)	Selection of all energy predictions between ΔΔG = 3–8.5 kcal mol^−1^	23	6	No correlation between T_1/2_ and ΔΔG_Foldx_ observed	1.9 Å	[112]
Protein-protein interaction prediction	SH2 domain (*Gallus gallus*)	Random sequences for binding	50,000	Area under the ROC curve 0.79 (accuracy)	FoldX can predict better than random binding events	2.1 Å	[113]
Improvement of DNA binding	Zinc finger nucleases (*Homo sapiens*)	Cut off value < ΔΔG −5 kcal mol^−1^ 420 predicted engineering sites Cut off value < ΔΔG −10 kcal mol^−1^	420	60% (low binding energy) 95% (high binding energy)	Improved DNA binding (−13 kcal mol^−1^)	1.6 Å	[96]
Protein stabilization	Anti-hVEGF antibody (*Homo sapiens*)	Single point mutations no cut off value reported	60	40% of tested sites were more stable	Stabilization (+ΔT_m_ = 2.2 °C (single site)) ΔT_m_ = 7 °C (combination)	Structure modeling	[86]
Destabilization of salt bridges	Subtilisin-like proteinase (*Thermus aquaticus*)	Salt bridge amino acids were mutated to Phe, Gln, Asn, Glu. FoldX calculation were performed 5 times and averaged for each mutation.	8	6	Highest destabilization (−ΔT_m_ = −8.8 °C)	1.55 Å	[114]
Protein stabilization	Growth factor 2 (*Homo sapiens*)	Cut off (ΔΔG < −1 kcal mol^−^1)	5	2	Stabilization ΔT_m_ = 3.7 °C	1.6 Å	[115]
	Flavin mononucleotide based fluorescent Protein (*Bacillus subtilis*)	Cut off (ΔΔG < −1 kcal mol^−1^) performed additionally further pre-selections	22	15	Stabilization ΔT_m_ = 11.4 °C	Resolution under 2.2 Å	[116]
	Endolysin PlyC (*Bacteriophage*)	Cut off (ΔΔG < −1 kcal mol^−1^) 92 mutants were determined by FoldX and reduced by visual inspection and by Rosetta	12	3	Stabilization ΔT_m_ = 2.2 °C	3.3 Å refined using Rosetta Relax	[117]
	*Database* (known mutations)	Cut off value ΔΔG < −1.0 kcal mol^−1^	30	20	n.d.	1.5 to 2.25 Å	[87]
Protein destabilization	Repair protein MSH2 (*Homo sapiens*)	Cut off value (ΔΔG > 5 kcal mol^−1^)	24	22	Destabilization of >3 kcal mol^−1^	3.3 Å	[85]
	*cblA*-Type methylmalonic aciduria (*Homo sapiens*)	Cut off value ΔΔG between 3.48 and 11.15 kcal mol^−1^	22	22	–	2.64 Å	[118]
Investigation of proline influence on stability	Fungal chimeric cellobiohydrolase Cel6A (*Humicola insolens*)	ΔΔG of exchanges of wild type amino acid against Pro exchange was calculated	17	57% were predicted correctly as destabilizing 43% as stabilizing	Destabilization ΔT_m_ = −4 °C Stabilization ΔT_m_ = +4 °C	1.3 Å	[119]
Influence of core residue substitutions on stability	Glycoside-hydrolase (*Neisseria polysaccharea*)	Settings not reported. 133 (7 sites) mutants were investigated towards stabilization or destabilization	57 (stabilizing) 76 (rest)	9 (stabilizing) 24 (destabilizing)	Stabilizing up to ΔT_m_ = 2° Destabilizing ΔT_m_ = − 6 °C	1.4 Å	[120]
Validation of estimations using FoldX	Laccase (*T. versicolor*)	2 sites selected as targets for stability in ionic liquid	2	2	1 variant showed stability improvement in ionic liquid	2.4 Å	[90]

### 2.3 Accuracy of FoldX

From the FoldX studies summarized in Table 1 it can be deduced that the crystal-structure quality is crucial for accurate calculations. From a benchmark test on myoglobin mutants Kepp concluded that some protein stability predicting algorithms are extremely sensitive towards crystal structure quality and some are very robust [121]. It seems plausible that interactions are in the order of atomic resolutions and therefore the crystal structure quality has an important influence on energy calculations [107,[121], [122], [123]]. However, for the prediction algorithms PoPmuSic, I-Mutant 3.0 and other tools the influence of the crystal structure quality was only in the order of 0.2 kcal mol^−1^ (standard deviation using different structure data of superoxide dismutase 1) [123].According to Christensen et al. FoldX belongs to the more structure sensitive methods and Kepp suggested to use only structures solved in scales of near-atomic-resolutions [107,121]. With reference to Table 1, all cited studies were based on crystal structures with an resolution better than 3.3 Å and an average resolution of 1.87 Å which is nearby atomic resolution (1 Å is approximately the diameter of an atom plus the surrounding cloud of electrons). Furthermore, also protein-protein interactions might have an influence on the prediction power, which are not addressed in some performance studies like from Tokuriki et al., because only monomeric proteins were selected [124], but e.g. Pey et al. and Dourado and Flores showed that also oligomers can be utilized for calculations (using extra terms: ΔG_kon_ electrostatic interaction, ΔS_tr_ translational and rotational entropy) [125,75]. The root-mean-square deviation (RMSD) in a dataset of protein complexes, with known energy impacts, was determined to be 1.55 kcal mol^−1^ (for single mutants) [75]. In contrast the algorithm ZEMu addresses such mutations on interfaces better than FoldX [75].

Based on experimental results, it can be concluded that the prediction of destabilizing mutations is more accurate than prediction of stabilizing mutations. After pre-selection of experiments with the aim to increase stability, it can be concluded that the approximate success rate for mutations predicted as stabilizing (according to their negative ΔΔG-values) is only 29.4% (focusing on 13 single mutation experiments). For experiments with focus on detection of destabilizing mutations or for simple proof of destabilizing events, sample size is only five but the average success rate is 69%. However, with regard to the small sample size a valid statement about success rates cannot be made. It is likely that many unsuccessful experiments were not published and therefore, the real success rate might be much lower. Khan et al. evaluated the performance of 11 protein stability predictors by using a dataset containing >1700 mutations in 80 proteins which were taken from ProTherm database. It was shown that FoldX was among the three most reliable algorithms, predicting 86 true positives and 133 false positives for stabilization from 776 variants, which is a success rate of 64%. Only Dmutant and MultiMutate were comparably successful in predicting stabilization events [102].

Compared to other results, this success rate might be higher than expected. As an example for an investigation of the performance of an adapted FoldX algorithm, laccase isoenzymes were used. The large calculation setup included 9424 FoldX predictions per isoenzyme using an adapted algorithm. These calculations were evaluated by using molecular dynamic simulations and additional different settings within FoldX were tested. Like mentioned before, the authors remarked that FoldX needs high-resolution crystal structures of proteins and that FoldX performs well in predicting stability trends, but not in a quantitative accuracy [75]. Using the deciphering protein (DPP) as an example, Kumar et al. showed on the basis of 54 DPP mutants how accurate the prediction power of FoldX is compared to other tools. The study focused on destabilizing mutation events, which were described in medical data sets of DPP and concluded that the R-value (correlation coefficient) was only 0.45 to 0.53. The quality of the crystal structures in this study ranged between 1.07 and 1.93 Å [77]. Potapov et al. utilized for performance investigation a protein database set regarding 2156 variants in 59 proteins. The crystal structure qualities were not reported. However, they concluded that 81.4% of T_m_ changes were qualitatively predicted correctly [127].

Furthermore Potapov et al. headlined their work for analyzing different protein stability tools “*Assessing computational methods for predicting protein stability upon mutation*: *good on average but not in the details*”, and proved that FoldX has potential to predict if a certain mutation is stabilizing or destabilizing, but its prediction power decreases, when ΔΔ*G* is correlated with ΔΔG_experimental_ or with stability parameters like T_m_ [127]. The correlation coefficient R, plotting ΔΔG_theoretical_ against ΔΔG_experimental_ values from databases was only 0.5 (for negative and positive ΔΔG), but it also depends on the crystal structure and on the nature of the protein [127].

For better comparison, we summarized statistical parameters given for the different algorithms derived from Kumar et al., and other studies (as indicated) in Table 2, but not for every algorithm we were able to find a full set of data. For example, Kumar et al. analyzed the predictive power of eight different tools, i.e. PoPMuSiC 3.1, BeatMuSiC, CUPSAT, I-Mutant 2.0/3.0, mCSM, ENCoM and FoldX, using the example of the human superoxide dismutase1 [73] which is involved in the motor neuron disease [131]. In this benchmark test FoldX and PoPMuSiC performed best by far. FoldX showed in this test a correlation coefficient R of 0.53 and a standard error of 1.1 kcal mol^−1^, which was only slightly surpassed by PoPMuSiC [77]. In conclusion, the authors described FoldX as more sensitive and accurate towards difficult mutation sites but PoPMuSiC as more accurate to all kinds of mutations. Also, they demonstrated that FoldX can interpret patient data for dismutase diseases quite well with an R of 0.45 compared to other tools. Bednar et al. compared FoldX with Rosetta-ddG, ERIS and CUPSAT [87] and determined FoldX and Rosetta-ddG as best algorithms for improving stability. Foit et al. showed for the immunity protein 7 that FoldX was able to predict destabilizing mutations very well (Coefficient of determination (R^2^) of 0.62), but the algorithm was unable to predict the influence of stabilizing mutants. However in comparison to I-Mutant 2.0, PoPMuSiC and Eris, FoldX showed a better performance for prediction of destabilizing mutagenesis events (R^2^-values of: 0.34, 0.24, 0.3) [132]. Tian et al. and by Broom et al. determined the R-value of FoldX with known true positives and true negatives to be 0.5 with an accuracy of 0.67 [98,95]. Ayuso-Tejedor et al. determined R-value with 0.20 to 0.29 for the corresponding mutants against predicted negative ΔΔG-values [130]. In contrast, by investigating 582 mutants of seven proteins, R was 0.73 with a standard deviation of 1.02 kcal mol^−1^ [134]. The best result was a correlation coefficient of 0.73 for a lysozyme structure [127] and was increased to 0.74, when only hotspot areas were chosen for prediction. The standard deviation (1.37 kcal mol^−1^) was in the same range of Broom et al. (1.78 kcal mol^−1^) [95]. However, Tokuriki et al. calculated that the average ΔΔG for any protein is +0.9 kcal mol^−1^ ΔΔG, which clearly shows, that the probability of destabilization events is much higher, which concludes that the number of stabilizing theoretical mutants is much lower [135]. Not only the number of theoretical stabilizing mutations seems to be lower, also the correlation for predicted stabilizing mutations towards real stabilization is weaker than for destabilizing mutations [57,111]. In contrast, Khan et al. showed for human proteins that FoldX predicts more stability increasing variants than destabilizing variants, which might be a hint that human proteins are relatively non-rigid and less thermostable compared to other protein sources or that distribution of ΔΔG_calculated_ against the frequency of stabilizing and destabilizing mutations is only protein depending [102]. Furthermore, the calculated ΔΔG_Foldx_ energies deviate from real ΔΔG measurements. The values can be recalculated using an experimental factor ΔΔG_experimental_ = (ΔΔG_calculated_ + 0.078) 1.14^−1^ [135,136]. Depending on the method used to evaluate FoldX, the accuracy will be in the range from 0.38 to 0.80 [102,129]. Obviously, FoldX can predict positions which are important for stability, but the discrimination between different amino acid residues at one site is not really powerful, e.g. an exchange of lysine to glutamate did not result in any change of ΔG, but experimentally a stabilization was observed [120,128]. The summarized results in Table 2 demonstrate that actually all algorithms are not able to design or predict single mutation events towards trustworthy one mutation protein designs. However, FoldX shows a good performance in most of the studies compared to other algorithms, but it is necessary to increase the number of experimental mutations above 3 to achieve probable true positive results for protein engineering experiments. A general disadvantage of FoldX and other algorithms seems to be that FoldX often predicts hydrophobic interactions but at the expense of protein solubility [95].

**Table 2:** Summary of different algorithms evaluated in performance tests considering prediction accuracy in comparison to experimentally investigated mutations and calculated statistical parameters. This table displays reported standard deviations of predicted true positives and true negatives. Accuracy is defined as ratio of true positives/true negatives to the total number of predictions. R-values (correlation coefficients) describe how precisely the predicted energies fit to database values.

Algorithm	Standard deviation	Accuracy range (min.–max.)	R-values
FoldX	1.0 to 1.78 kcal mol^−1^ [118,116,95]	0.38 to 0.8 [128] Average accuracy: 0.69 [87,103,127,129]	0.29 [130] to 0.73 [118]
BeatMuSiC	1.2 kcal mol^−1^ [77]		0.46 [77]
CUPSAT	1.8 kcal mol^−1^ [77]	0.5 [102]	0.3 [77]
I-Mutant 2.0/3.0	1.2 to 1.52 kcal mol^−1^ [77,95]	0.48 [102] to 0.75 [127]	0.16 [77] to 0.51 [95]
PoPMuSiC	1.1 kcal mol^−1^ to 1.32 [77,95]	0.62 [129] to 0.85 [129]	0.51 to 0.55 [95,77]
mCSM	3.2 kcal mol^−1^ [77]		0.23 [77]
ENCoM	1.5 kcal mol^−1^ [77]		0.04 [77]
Rosetta-ddG	2.3 kcal mol^−1^ [95]	0.71 [127] to 0.76 [87]	0.26 [127] to 0.54 [95]

### 2.4 The Next Generation of FoldX Based Predictions

Due to the low accuracy of all algorithms for stabilization mutations, algorithms often are combined to find coincident predictions or to prove predictions with a second algorithm. A popular combination is FoldX and Rosetta-ddG to gain more stabilizing mutation predictions. It was shown that FoldX and Rosetta-ddG predictions overlapped only in 12%, 15% or 25%, respectively, which means that a good coverage of beneficial mutations can only be achieved when more than one tool is used [57,87,105]. As a consequence of low prediction accuracy, popular algorithms are continuously improved. Recently a refinement of the Rosetta energy algorithm was reported with increased accuracy and faster calculation times. This demonstrates also the continuing importance of stability prediction in the field of protein engineering, but the authors stated that it is still far away from a final gold standard in the field of energy content prediction [137].

A sophisticated approach is the freeware webtool FireProt [138]. The FireProt algorithm uses FoldX as a pre-filter to select beneficial mutations which are subsequently proved in a second round using Rosetta-ddG. Only if Rosetta-ddG also predicts these mutations as putatively stabilizing they will be used for the experimental realization of these amino acid exchanges. Furthermore, the algorithm uses a consensus analysis of the protein-sequences to predict evolutionary beneficial mutation sites towards stability. These selected sites are then evaluated for their suitability using FoldX. The algorithm is divided into three stages using different methods for crosschecking the accuracy of the calculations and combines putative beneficial mutations to gain further improvement of stability. The free webtool of FireProt allows even inexperienced users to perform protein energy calculations. Bednar et al. demonstrated at two examples the utility of this algorithm using the example of two enzymes and combined many mutation sites with overall improvements of ΔT_m_ of 21 °C and 24 °C for the combination of all sites [87]. However, to verify if FireProt is useful or not, more studies are necessary. Furthermore, the core function of FoldX algorithm does not simulate backbone movements of the protein, which might be a potential factor to improve FoldX [75]. The stability prediction tool of Goldenzweig et al. might be an alternative to the mentioned Fireprot -algorithm. Similar to Fireprot, it combines information gained in sequence homology alignments and of energy calculations using crystal structure data and Rosetta-ddG. Using the human acetylcholinesterase, an improvement in stability (ΔT_m_ = 20 °C) was demonstrated and simultaneously, the expression level in *E. coli* BL21 was increased. They hypothesized, that putatively destabilizing mutations can be excluded from mutation libraries using homologous sequence alignments to prohibit certain types of amino acid exchanges [139].

## 3 Conclusion

The performance of FoldX depends drastically on the quality of the crystal structure and it is unclear if the protein source might have an influence on the accuracy of such algorithms. Nevertheless, FoldX seems to be more accurate for the prediction of destabilizing mutations and less accurate for the prediction of stabilizing mutations, but in both cases it was shown that FoldX is clearly better than random approaches: e.g. Christensen et al. described FoldX as one of the most accurate single site stability predictors and Potapov et al. even described the accuracy of FoldX as impressive compared to other algorithms [122,127]. The natural success rate for random mutagenesis is only ~2%, which was surpassed by most experiments [95,140]. Therefore, FoldX seems to be a promising tool for protein design, but as mentioned by Thiltgen et al. we agree that FoldX cannot serve as a gold-standard for generally improving stability of proteins. Moreover, using FoldX together with other algorithms for reciprocal control of calculation results, Rosetta-ddG or PoPmuSiC as filter for true positive results will most probably increase the accuracy and the success rate of thermostability engineering [141,87,95]. In general the accuracy can be improved additionally, when mutation outliers are eliminated or additional MD-simulations are performed [83]. FoldX was used successfully in different approaches (Table 1) aiming from enzyme stabilization towards predictions of protein-protein interactions (especially for drug design) or for the prediction of disease-associated mutant proteins, making FoldX a versatile tool for life science [81,[142], [143], [144]].The progress in protein stability prediction is striking, however up to now no in silico calculation can fully spare experimental procedures, although the existing tools can reduce the amount of lab experiments significantly.

## References

[1] Newman J.D., Turner A.P.F. (2005). Home blood glucose biosensors: a commercial perspective. Biosens Bioelectron.

[2] Capdevila J., Elez E., Macarulla T., Ramos F.J., Ruiz-Echarri M., Tabernero J. (2009). Anti-epidermal growth factor receptor monoclonal antibodies in cancer treatment. Cancer Treat Rev.

[3] Bhosale S.H., Rao M.B., Deshpande V.V. (1996). Molecular and industrial aspects of glucose isomerase. Microbiol Rev.

[4] Savile C.K., Janey J.M., Mundorff E.C., Moore J.C., Tam S., Jarvis W.R. (2010). Biocatalytic asymmetric synthesis of chiral amines from ketones applied to sitagliptin manufacture. Science.

[5] Pollard D.J., Woodley J.M. (2007). Biocatalysis for pharmaceutical intermediates: the future is now. Trends Biotechnol.

[6] Mittal S., Kaur H., Gautam N., Mantha A.K. (2017). Biosensors for breast cancer diagnosis: a review of bioreceptors, biotransducers and signal amplification strategies. Biosens Bioelectron.

[7] Yoo E.-H., Lee S.-Y. (2010). Glucose biosensors: an overview of use in clinical practice. Sensors.

[8] Sin M.L., Mach K.E., Wong P.K., Liao J.C. (2014). Advances and challenges in biosensor-based diagnosis of infectious diseases. Expert Rev Mol Diagn.

[9] Willuda J., Honegger A., Waibel R., Schubiger P.A., Stahel R., Zangemeister-Wittke U. (1999). High thermal stability is essential for tumor targeting of antibody fragments. Cancer Res.

[10] Klein-Marcuschamer D., Oleskowicz-Popiel P., Simmons B.A., Blanch H.W. (2012). The challenge of enzyme cost in the production of lignocellulosic biofuels. Biotechnol Bioeng.

[11] Sheldon R.A., van Pelt S. (2013). Enzyme immobilisation in biocatalysis: why, what and how. Chem Soc Rev.

[12] Yazbeck D.R., Martinez C.A., Hu S., Tao J. (2004). Challenges in the development of an efficient enzymatic process in the pharmaceutical industry. Tetrahedron Asymmetry.

[13] Woodley J.M. (2013). Protein engineering of enzymes for process applications. Curr Opin Chem Biol.

[14] Salihu A., Alam M.Z. (2015). Solvent tolerant lipases: a review. Process Biochem.

[15] Peterson M.E., Daniel R.M., Danson M.J., Eisenthal R. (2007). The dependence of enzyme activity on temperature: determination and validation of parameters. Biochem J.

[16] Daniel R.M., Peterson M.E., Danson M.J., Price N.C., Kelly S.M., Monk C.R. (2010). The molecular basis of the effect of temperature on enzyme activity. Biochem J.

[17] Jones H.B.L., Wells S.A., Prentice E.J., Kwok A., Liang L.L., Arcus V.L. (2017). A complete thermodynamic analysis of enzyme turnover links the free energy landscape to enzyme catalysis. FEBS J.

[18] Reyes B.A., Pendergast J.S., Yamazaki S. (2008). Mammalian peripheral circadian oscillators are temperature compensated. J Biol Rhythm.

[19] Rejasse B., Maugard T., Legoy M.D. (2003). Enzymatic procedures for the synthesis of water-soluble retinol derivatives in organic media. Enzym Microb Technol.

[20] Laidler K.J., Peterman B.F. (1979). Temperature effects in enzyme kinetics. Methods Enzymol.

[21] Wolfenden R., Snider M., Ridgway C., Miller B. (1999). The temperature dependence of enzyme rate enhancements. J Am Chem Soc.

[22] Vieille C., Zeikus G.J. (2001). Hyperthermophilic enzymes: sources, uses, and molecular mechanisms for thermostability. Microbiol Mol Biol Rev.

[23] Daniel R.M., Danson M.J., Eisenthal R. (2001). The temperature optima of enzymes: a new perspective on an old phenomenon. Trends Biochem Sci.

[24] Salazar O., Cirino P.C., Arnold F.H. (2003). Thermostabilization of a cytochrome P450 peroxygenase. ChemBioChem.

[25] Han Z.L., Han S.Y., Zheng S.P., Lin Y. (2009). Enhancing thermostability of a Rhizomucor miehei lipase by engineering a disulfide bond and displaying on the yeast cell surface. Appl Microbiol Biotechnol.

[26] Takagi H., Takahashi T., Momose H., Inouye M., Maeda Y., Matsuzawa H. (1990). Enhancement of the thermostability of subtilisin E by introduction of a disulfide bond engineered on the basis of structural comparison with a thermophilic serine protease. J Biol Chem.

[27] Kim J., Grate J.W., Wang P. (2006). Nanostructures for enzyme stabilization. Chem Eng Sci.

[28] Carninci P., Nishiyama Y., Westover A., Itoh M., Nagaoka S., Sasaki N. (1998). Thermostabilization and thermoactivation of thermolabile enzymes by trehalose and its application for the synthesis of full length cDNA. Proc Natl Acad Sci U S A.

[29] Korkegian A., Black M.E., Baker D., Stoddard B.L. (2005). Computational thermostabilization of an enzyme. Science.

[30] Miyazaki K., Arnold F.H. (1999). Exploring nonnatural evolutionary pathways by saturation mutagenesis: rapid improvement of protein function. J Mol Evol.

[31] Miyazaki K., Wintrode P.L., Grayling R.A., Rubingh D.N., Arnold F.H. (2000). Directed evolution study of temperature adaptation in a psychrophilic enzyme. J Mol Biol.

[33] Karshikoff A., Nilsson L., Ladenstein R. (2015). Rigidity versus flexibility: the dilemma of understanding protein thermal stability. FEBS J.

[34] Lee G.M., Palsson B.O. (1990). Immobilization can improve the stability of hybridoma antibody productivity in serum-free media. Biotechnol Bioeng.

[35] Radadia A.D., Stavis C.J., Carr R., Zeng H., King W.P., Carlisle J.A. (2011). Control of nanoscale environment to improve stability of immobilized proteins on diamond surfaces. Adv Funct Mater.

[36] Dold S.-M., Cai L., Rudat J. (2016). One-step purification and immobilization of a β-amino acid aminotransferase using magnetic (M-PVA) beads. Eng Life Sci.

[37] Dinçer A., Telefoncu A. (2007). Improving the stability of cellulase by immobilization on modified polyvinyl alcohol coated chitosan beads. J Mol Catal B Enzym.

[38] Li D., He Q., Cui Y., Duan L., Li J. (2007). Immobilization of glucose oxidase onto gold nanoparticles with enhanced thermostability. Biochem Biophys Res Commun.

[39] Matsumoto M., Ohashi K. (2003). Effect of immobilization on thermostability of lipase from *Candida rugosa*. Biochem Eng J.

[40] De Cordt S., Vanhoof K., Hu J., Maesmans G., Hendrickx M., Tobback P. (1992). Thermostability of soluble and immobilized α-amylase from *Bacillus licheniformis*. Biotechnol Bioeng.

[41] Liu B.L., Jong C.H., Tzeng Y.M. (1999). Effect of immobilization on pH and thermal stability of *Aspergillus ficuum* phytase. Enzym Microb Technol.

[42] Lülsdorf N., Vojcic L., Hellmuth H., Weber T.T., Mußmann N., Martinez R. (2015). A first continuous 4-aminoantipyrine (4-AAP)-based screening system for directed esterase evolution. Appl Microbiol Biotechnol.

[43] Midelfort K.S., Kumar R., Han S., Karmilowicz M.J., McConnell K., Gehlhaar D.K. (2013). Redesigning and characterizing the substrate specificity and activity of *Vibrio fluvialis* aminotransferase for the synthesis of imagabalin. Protein Eng Des Sel.

[44] Lauchli R., Rabe K.S., Kalbarczyk K.Z., Tata A., Heel T., Kitto R.Z. (2013). High-throughput screening for terpene-synthase-cyclization activity and directed evolution of a terpene synthase. Angew Chem Int Ed.

[45] Giver L., Gershenson A., Freskgard P.O., Arnold F.H. (1998). Directed evolution of a thermostable esterase. Proc Natl Acad Sci U S A.

[46] Lehmann M., Wyss M. (2001). Engineering proteins for thermostability: the use of sequence alignments versus rational design and directed evolution. Curr Opin Biotechnol.

[47] Eijsink V.G.H., GÅseidnes S., Borchert T.V., Van Den Burg B. (2005). Directed evolution of enzyme stability. Biomol Eng.

[48] Zhou C., Ye J., Xue Y., Ma Y. (2015). Directed evolution and structural analysis of alkaline pectate lyase from the alkaliphilic bacterium *Bacillus* sp. strain N16-5 to improve its thermostability for efficient ramie degumming. Appl Environ Microbiol.

[49] Wörn A., Plückthun A. (2001). Stability engineering of antibody single-chain Fv fragments. J Mol Biol.

[50] Famm K., Hansen L., Christ D., Winter G. (2008). Thermodynamically stable aggregation-resistant antibody domains through directed evolution. J Mol Biol.

[51] Van Durme J., Delgado J., Stricher F., Serrano L., Schymkowitz J., Rousseau F. (2011). A graphical interface for the FoldX forcefield. Bioinformatics.

[52] Bunting K.A., Cooper J.B., Tickle I.J., Young D.B. (2002). Engineering of an intersubunit disulfide bridge in the iron-superoxide dismutase of *Mycobacterium tuberculosis*. Arch Biochem Biophys.

[53] Dehnavi E., Fathi-Roudsari M., Mirzaie S., Arab S.S., Ranaei Siadat S.O., Khajeh K. (2017). Engineering disulfide bonds in *Selenomonas ruminantium* β-xylosidase by experimental and computational methods. Int J Biol Macromol.

[54] Kabashima T., Li Y., Kanada N., Ito K., Yoshimoto T. (2001). Enhancement of the thermal stability of pyroglutamyl peptidase I by introduction of an intersubunit disulfide bond. Biochim Biophys Acta Protein Struct Mol Enzymol.

[55] Matsumurat M., Becktelt W.J., Levitr M., Matthewstl B.W. (1989). Stabilization of phage T4 Iysozyme by engineered disulfide bonds (thermostability/lysozyme/protein structure). Biochemistry.

[56] Dombkowski A.A., Sultana K.Z., Craig D.B. (2014). Protein disulfide engineering. FEBS Lett.

[57] Wijma H.J., Floor R.J., Jekel P.A., Baker D., Marrink S.J., Janssen D.B. (2014). Computationally designed libraries for rapid enzyme stabilization. Protein Eng Des Sel.

[58] Dombkowski A.A. (2003). Disulfide by Design™: a computational method for the rational design of disulfide bonds in proteins. Bioinformatics.

[59] Craig D.B., Dombkowsk A.A. (2013). Disulfide by Design 2.0: a web-based tool for disulfide engineering in proteins. BMC Bioinf.

[60] Jo B.H., Park T.Y., Park H.J., Yeon Y.J., Yoo Y.J., Cha H.J. (2016). Engineering de novo disulfide bond in bacterial α-type carbonic anhydrase for thermostable carbon sequestration. Sci Rep.

[61] Vlassi M., Cesareni G., Kokkinidis M. (1999). A correlation between the loss of hydrophobic core packing interactions and protein stability. Edited by A. R. Fersht. J Mol Biol.

[62] Leaver-Fay A., Tyka M., Lewis S.M., Lange OF, Thompson J., Jacak R. (2011). ROSETTA3: an object-oriented software suite for the simulation and design of macromolecules. Methods Enzymol.

[63] Akasako A., Haruki M., Oobatake M., Kanaya S. (1997). Conformational stabilities of *Escherichia coli* RNase HI variants with a series of amino acid substitutions at a cavity within the hydrophobic core. J Biol Chem.

[64] Borgo B., Havranek J.J. (2012). Automated selection of stabilizing mutations in designed and natural proteins. Proc Natl Acad Sci.

[65] Lee T.M. (2014). Computationally-guided thermostabilization of the primary endoglucanase from *Hypocrea jecorina* for cellulosic biofuel production.

[66] Marshall S.A., Morgan C.S., Mayo S.L. (2002). Electrostatics significantly affect the stability of designed homeodomain variants. J Mol Biol.

[67] Steipe B., Schiller B., Plückthun A., Steinbacher S. (1994). Sequence statistics reliably predict stabilizing mutations in a protein domain. J Mol Biol.

[68] Wang Q., Buckle A.M., Foster N.W., Johnson C.M., Fersht A.R. (1999). Design of highly stable functional GroEL minichaperones. Protein Sci.

[69] Anbar M., Gul O., Lamed R., Sezerman U.O., Bayer E.A. (2012). Improved thermostability of *Clostridium thermocellum* endoglucanase Cel8A by using consensus-guided mutagenesis. Appl Environ Microbiol.

[70] Komor R.S., Romero P.A., Xie C.B., Arnold F.H. (2012). Highly thermostable fungal cellobiohydrolase i (Cel7A) engineered using predictive methods. Protein Eng Des Sel.

[71] Huang L., Xu J.H., Yu H.L. (2015). Significantly improved thermostability of a reductase CgKR1 from *Candida glabrata* with a key mutation at Asp 138 for enhancing bioreduction of aromatic α-keto esters. J Biotechnol.

[72] Parthiban V., Gromiha M.M., Schomburg D. (2006). CUPSAT: prediction of protein stability upon point mutations. Nucleic Acids Res.

[73] Modarres H.P., Mofrad M.R., Sanati-Nezhad A. (2016). Protein thermostability engineering. RSC Adv.

[74] Panigrahi P., Sule M., Ghanate A., Ramasamy S., Suresh C.G. (2015). Engineering proteins for thermostability with iRDP web server. PLoS One.

[75] Dourado D.F.A.R., Flores S.C. (2014). A multiscale approach to predicting affinity changes in protein-protein interfaces. Proteins Struct Funct Bioinf.

[76] Bava K.A., Gromiha M.M., Uedaira H., Kitajima K., Sarai A. (2004). ProTherm, version 4.0: thermodynamic database for proteins and mutants. Nucleic Acids Res.

[77] Kumar V., Rahman S., Choudhry H., Zamzami M.A., Sarwar Jamal M., Islam A. (2017). Computing disease-linked SOD1 mutations: deciphering protein stability and patient-phenotype relations. Sci Rep.

[78] Dehouck Y., Kwasigroch J.M., Rooman M., Gilis D. (2013). BeAtMuSiC: prediction of changes in protein-protein binding affinity on mutations. Nucleic Acids Res.

[79] Frappier V., Chartier M., Najmanovich R.J. (2015). ENCoM server: exploring protein conformational space and the effect of mutations on protein function and stability. Nucleic Acids Res.

[80] Reetz M.T., Carballeira J.D., Vogel A. (2006). Iterative saturation mutagenesis on the basis of b factors as a strategy for increasing protein thermostability. Angew Chem Int Ed.

[81] Buß O., Muller D., Jager S., Rudat J., Rabe K.S. (2017). Improvement of the thermostability of a β-amino acid converting ω-transaminase using FoldX. ChemBioChem.

[82] Guerois R., Nielsen J.E., Serrano L. (2002). Predicting changes in the stability of proteins and protein complexes: a study of more than 1000 mutations. J Mol Biol.

[83] Schymkowitz J., Borg J., Stricher F., Nys R., Rousseau F., Serrano L. (2005). The FoldX web server: an online force field. Nucleic Acids Res.

[84] Grulich M., Brezovský J., Štěpánek V., Palyzová A., Marešová H., Zahradník J. (2016). In-silico driven engineering of enantioselectivity of a penicillin G acylase towards active pharmaceutical ingredients. J Mol Catal B Enzym.

[85] Nielsen S.V., Stein A., Dinitzen A.B., Papaleo E., Tatham M.H., Poulsen E.G. (2017). Predicting the impact of Lynch syndrome-causing missense mutations from structural calculations. PLoS Genet.

[86] Wang S., Liu M., Zeng D., Qiu W., Ma P., Yu Y. (2014). Increasing stability of antibody via antibody engineering: stability engineering on an anti-hVEGF. Proteins Struct Funct Bioinf.

[87] Bednar D., Beerens K., Sebestova E., Bendl J., Khare S., Chaloupkova R. (2015). FireProt: energy- and evolution-based computational design of thermostable multiple-point mutants. PLoS Comput Biol.

[88] Bhaskara R.M., Srinivasan N. (2011). Stability of domain structures in multi-domain proteins. Sci Rep.

[89] Bhaskara R.M., De Brevern A.G., Srinivasan N. (2013). Understanding the role of domain-domain linkers in the spatial orientation of domains in multi-domain proteins. J Biomol Struct Dyn.

[90] Liu H., Zhu L., Bocola M., Chen N., Spiess A.C., Schwaneberg U. (2013). Directed laccase evolution for improved ionic liquid resistance. Green Chem.

[91] Alibés A., Serrano L., Nadra A.D. (2010). Structure-based DNA-binding prediction and design. Methods Mol Biol.

[92] Wahab R.A., Basri M., Rahman M.B.A., Rahman R.N.Z.R.A., Salleh A.B., Chor L.T. (2012). Engineering catalytic efficiency of thermophilic lipase from *Geobacillus zalihae* by hydrophobic residue mutation near the catalytic pocket. Adv Biosci Biotechnol.

[93] Wahab R.A., Basri M., Rahman R.N.Z.R.A., Salleh A.B., Rahman M.B.A., Chor L.T. (2012). Manipulation of the conformation and enzymatic properties of T1 lipase by site-directed mutagenesis of the protein core. Appl Biochem Biotechnol.

[94] Fernandez-Escamilla A.-M., Rousseau F., Schymkowitz J., Serrano L. (2004). Prediction of sequence-dependent and mutational effects on the aggregation of peptides and proteins. Nat Biotechnol.

[95] Broom A., Jacobi Z., Trainor K., Meiering E.M. (2017). Computational tools help improve protein stability but with a solubility tradeoff. J Biol Chem.

[96] He Z., Mei G., Zhao C., Chen Y. (2011). Potential application of FoldX force field based protein modeling in zinc finger nucleases design. Sci China Life Sci.

[97] Szczepek M., Brondani V., Büchel J., Serrano L., Segal D.J., Cathomen T. (2007). Structure-based redesign of the dimerization interface reduces the toxicity of zinc-finger nucleases. Nat Biotechnol.

[98] Tian J., Wu N., Chu X., Fan Y. (2010). Predicting changes in protein thermostability brought about by single- or multi-site mutations. BMC Bioinf.

[99] Arabnejad H., Dal Lago M., Jekel P.A., Floor R.J., Thunnissen A.-M.W.H., Terwisscha van Scheltinga A.C. (2016). A robust cosolvent-compatible halohydrin dehalogenase by computational library design. Protein Eng Des Sel.

[100] Parker A.S., Zheng W., Griswold K.E., Bailey-Kellogg C. (2010). Optimization algorithms for functional deimmunization of therapeutic proteins. BMC Bioinf.

[101] Li M., Simonetti F.L., Goncearenco A., Panchenko A.R. (2016). MutaBind estimates and interprets the effects of sequence variants on protein-protein interactions. Nucleic Acids Res.

[102] Khan S., Vihinen M. (2010). Performance of protein stability predictors. Hum Mutat.

[103] Luo X.J., Zhao J., Li C.X., Bai Y.P., Reetz M.T., Yu H.L. (2016). Combinatorial evolution of phosphotriesterase toward a robust malathion degrader by hierarchical iteration mutagenesis. Biotechnol Bioeng.

[104] Timucin E., Sezerman O.U. (2013). The conserved lid tryptophan, w211, potentiates thermostability and thermoactivity in bacterial thermoalkalophilic lipases. PLoS One.

[105] Floor R.J., Wijma H.J., Colpa D.I., Ramos-Silva A., Jekel P.A., Szymański W. (2014). Computational library design for increasing haloalkane dehalogenase stability. ChemBioChem.

[106] Huang J., Xie D.F., Feng Y. (2017). Engineering thermostable (R)-selective amine transaminase from aspergillus terreus through in silico design employing B-factor and folding free energy calculations. Biochem Biophys Res Commun.

[107] Christensen N.J., Kepp K.P. (2012). Accurate stabilities of laccase mutants predicted with a modified FoldX protocol. J Chem Inf Model.

[108] Rahman R.N.Z.R.A., Zakaria I.I., Salleh A.B., Basri M. (2012). Enzymatic properties and mutational studies of chalcone synthase from *Physcomitrella* patens. Int J Mol Sci.

[109] Li M., Zhang Z.J., Kong X.D., Yu H.L., Zhou J., Xu J.H. (2017). Engineering *Streptomyces coelicolor* carbonyl reductase for efficient atorvastatin precursor synthesis. Appl Environ Microbiol.

[110] Wu B., Wijma H.J., Song L., Rozeboom H.J., Poloni C., Tian Y. (2016). Versatile peptide C-terminal functionalization via a computationally engineered peptide amidase. ACS Catal.

[111] Polizzi K.M., Chaparro-Riggers J.F., Vazquez-Figueroa E., Bommarius A.S. (2006). Structure-guided consensus approach to create a more thermostable penicillin G acylase. Biotechnol J.

[112] Sullivan B.J., Nguyen T., Durani V., Mathur D., Rojas S., Thomas M. (2012). Stabilizing proteins from sequence statistics: the interplay of conservation and correlation in triosephosphate isomerase stability. J Mol Biol.

[113] Sánchez I.E., Beltrao P., Stricher F., Schymkowitz J., Ferkinghoff-Borg J., Rousseau F. (2008). Genome-wide prediction of SH2 domain targets using structural information and the FoldX algorithm. PLoS Comput Biol.

[114] Jónsdóttir L.B., Ellertsson B., Invernizzi G., Magnúsdóttir M., Thorbjarnardóttir S.H., Papaleo E. (1844). The role of salt bridges on the temperature adaptation of aqualysin I, a thermostable subtilisin-like proteinase. Biochim Biophys Acta, Proteins Proteomics.

[115] Dvorak P., Bednar D., Vanacek P., Balek L., Eiselleova L., Stepankova V. (2016). Computer-assisted engineering of hyperstable fibroblast growth factor 2. bioRxiv.

[116] Song X., Wang Y., Shu Z., Hong J., Li T., Yao L. (2013). Engineering a more thermostable blue light photo receptor *Bacillus subtilis* YtvA LOV domain by a computer aided rational design method. PLoS Comput Biol.

[117] Heselpoth R.D., Yin Y., Moult J., Nelson D.C. (2015). Increasing the stability of the bacteriophage endolysin PlyC using rationale-based FoldX computational modeling. Protein Eng Des Sel.

[118] Plessl T., Bürer C., Lutz S., Yue W.W., Baumgartner M.R., Froese D.S. (2017). Protein destabilization and loss of protein-protein interaction are fundamental mechanisms in cblA-type methylmalonic aciduria. Hum Mutat.

[119] Wu I. (2013). Engineering thermostable fungal cellobiohydrolases.

[120] Daudé D., Topham C.M., Remaud-Siméon M., André I. (2013). Probing impact of active site residue mutations on stability and activity of *Neisseria polysaccharea* amylosucrase. Protein Sci.

[121] Kepp K.P. (1854). Towards a “golden standard” for computing globin stability: stability and structure sensitivity of myoglobin mutants. Biochim Biophys Acta, Proteins Proteomics.

[122] Christensen N.J., Kepp K.P. (2013). Stability mechanisms of laccase isoforms using a modified FoldX protocol applicable to widely different proteins. J Chem Theory Comput.

[123] Kepp K.P. (2014). Computing stability effects of mutations in human superoxide dismutase 1. J Phys Chem B.

[124] Tokuriki N., Stricher F., Schymkowitz J., Serrano L., Tawfik D.S. (2007). The stability effects of protein mutations appear to be universally distributed. J Mol Biol.

[125] Pey A.L., Stricher F., Serrano L., Martinez A. (2007). Predicted effects of missense mutations on native-state stability account for phenotypic outcome in phenylketonuria, a paradigm of misfolding diseases. Am J Hum Genet.

[127] Potapov V., Cohen M., Schreiber G. (2009). Assessing computational methods for predicting protein stability upon mutation: good on average but not in the details. Protein Eng Des Sel.

[128] Modarres P. (2015). Modeling and engineering proteins thermostability.

[129] Fleming N., Kinsella B., Ing C. (2016). Predicting protein thermostability upon mutation using molecular dynamics timeseries data. bioRxiv.

[130] Ayuso-Tejedor S., Abián O., Sancho J. (2011). Underexposed polar residues and protein stabilization. Protein Eng Des Sel.

[131] Valentine J.S., Doucette P.A., Zittin Potter S. (2005). Copper-zinc superoxide dismutase and amyotrophic lateral sclerosis. Annu Rev Biochem.

[132] Foit L., Morgan G.J., Kern M.J., Steimer L.R., von Hacht A.A., Titchmarsh J. (2009). Optimizing protein stability in vivo. Mol Cell.

[134] Benedix A., Becker C.M., de Groot B.L., Caflisch A., Böckmann R.A. (2009). Predicting free energy changes using structural ensembles. Nat Methods.

[135] Tokuriki N., Stricher F., Serrano L., Tawfik D.S. (2008). How protein stability and new functions trade off. PLoS Comput Biol.

[136] Sánchez I.E., Tejero J., Gómez-Moreno C., Medina M., Serrano L. (2006). Point mutations in protein globular domains: contributions from function, stability and misfolding. J Mol Biol.

[137] Alford R.F., Leaver-Fay A., Jeliazkov J.R., O'Meara M.J., DiMaio F.P., Park H. (2017). The Rosetta all-atom energy function for macromolecular modeling and design. J Chem Theory Comput.

[138] Musil M., Stourac J., Bendl J., Brezovsky J., Prokop Z., Zendulka J. (2017). FireProt: web server for automated design of thermostable proteins. Nucleic Acids Res.

[139] Goldenzweig A., Goldsmith M., Hill S.E., Gertman O., Laurino P., Ashani Y. (2016). Automated structure- and sequence-based design of proteins for high bacterial expression and stability. Mol Cell.

[140] Bromberg Y., Rost B., Kruglyak L., Nickerson D., Schmitt A., Schuchhardt J. (2009). Correlating protein function and stability through the analysis of single amino acid substitutions. BMC Bioinf.

[141] Thiltgen G., Goldstein R.A. (2012). Assessing predictors of changes in protein stability upon mutation using self-consistency. PLoS One.

[142] Bakail M., Ochsenbein F. (2016). Targeting protein–protein interactions, a wide open field for drug design. C R Chim.

[143] Schubert B., Schärfe C., Dönnes P., Hopf T., Marks D., Kohlbacher O. (2017). Population-specific design of de-immunized protein biotherapeutics. arXiv.

[144] Yue W.W. (2016). From structural biology to designing therapy for inborn errors of metabolism. J Inherit Metab Dis.

[145] Wijma H.J., Fürst M.J.L.J., Janssen D.B., Bornscheuer U, Höhne M (2018). A Computational Library Design Protocol for Rapid Improvement of Protein Stability: FRESCO. Protein Engineering.

